# MSM HIV testing following an online testing intervention in China

**DOI:** 10.1186/s12879-017-2546-y

**Published:** 2017-06-19

**Authors:** Ngai Sze Wong, Weiming Tang, Larry Han, John Best, Ye Zhang, Shujie Huang, Heping Zheng, Bin Yang, Chongyi Wei, Stephen W. Pan, Joseph D. Tucker

**Affiliations:** 1 University of North Carolina Project-China, Guangzhou, Guangdong China; 2 Institute for Global Health & Infectious Diseases, University of North Carolina at Chapel Hill, Chapel Hill, NC USA; 3 Stanley Ho Centre for Emerging Infectious Diseases, The Chinese University of Hong Kong, Hong Kong, China; 4 SESH Global, Guangzhou, Guangdong China; 5 Guangdong Provincial Centres for Skin Diseases and STI Control, Guangzhou, Guangdong China; 6 Department of Biostatistics, University of North Carolina at Chapel Hill, Chapel Hill, NC USA; 7 School of Medicine, University of California, San Francisco, CA USA; 8 Dermatology Hospital of Southern Medical University, Guangzhou, Guangdong China; 9 Department of Epidemiology and Biostatistics, University of California, San Francisco, CA USA

**Keywords:** HIV testing, Internet, China, MSM, Multilevel model

## Abstract

**Background:**

Scaling up HIV testing is the first step in the HIV treatment continuum which is important for controlling the HIV epidemic among men who have sex with men (MSM). Following an online HIV testing intervention among MSM, we aim to examine sociodemographic and spatial factors associated with HIV testing.

**Methods:**

We conducted a secondary analysis on data from an online HIV testing intervention among MSM who had never-tested for HIV. The survey was distributed through online networks connected to all provinces and regions of China. Univariate and multivariable analyses were performed to examine factors associated with testing three weeks post-intervention.

**Results:**

At three weeks after the intervention, 36% of 624 followed-up MSM underwent HIV testing, 69 men reported positive HIV test results. Having money for sex, ever tested for sexually transmitted infections and intimate partner violence experience were significant factors of post-intervention HIV testing. Students were less likely to undergo HIV testing at follow-up compared to others (adjusted odds ratio=0.69, 95% C.I.=0.47–0.99), adjusted by age and type of intervention. Moderate provincial spatial variation of testing was observed.

**Conclusions:**

While high risk men generally had higher HIV testing rates, some MSM like students had lower testing rates, suggesting the need for further ways to enhance HIV testing in specific MSM communities.

**Electronic supplementary material:**

The online version of this article (doi:10.1186/s12879-017-2546-y) contains supplementary material, which is available to authorized users.

## Background

HIV testing is the first step in the HIV care continuum [1]. Expanding HIV testing results in larger number of individuals diagnosed with HIV, thereby introducing more opportunities to avert HIV transmission. In China, HIV testing coverage among men who have sex with men (MSM) has been low, with only 24% ever tested in the years 2000–2007 [2]. HIV prevalence among MSM in China increased from 1% in 2003 to 7.7% in 2014 [3]. Due partly to the Chinese National Free Antiretroviral Treatment Program which began in 2003 (free testing, care and treatment) [4] and adoption of strategic plan targeting at MSM in 2007–2010, [5] the proportion of MSM who had ever undergone HIV testing increased to 47% by 2011 [2]. This is still far behind the UNAIDS target for 90% testing among infected individuals [6].

In the digital era, one way to enhance HIV testing is by disseminating HIV testing promotion messages through the internet. The internet provides an opportunity to deliver messages efficiently with fewer geographic constraints. MSM in China have high rates of internet and smart phone use, providing a foundation for online interventions [7]. HIV testing promotion using mass media such as short videos are commonly used to enhance HIV testing [8, 9]. The World Health Organization also advocated the use of media interventions to tailor HIV testing promotion among subgroups, especially key populations [10]. Most studies of mass media HIV testing interventions for MSM have been limited to high-income countries such as Australia, the United States and the United Kingdom [8, 11–13]. In view of high transmission risk of HIV among MSM in China, we examined sociodemographic and spatial factors associated with HIV testing following an online HIV testing intervention among never-tested MSM.

## Methods

This is a secondary analysis of data from an online intervention that focused on promoting HIV testing in China in 2014 (study details elsewhere [14, 15]) In brief, 1424 MSM had been recruited online through community-based organizations with web portals based throughout China. Men who had anal sex with men at least once during their lifetime, were ≥16 year-old, and had never-tested for HIV were included. After completing an online survey, 721 never-tested participants (after excluding 703 (49.4%) ever tested participants) were randomly assigned to view either a crowdsourced video or health marketing video (one minute) that promoted HIV testing. Crowdsourcing is a bottom-up approach that uses crowd (non-professionals) wisdom to create a product or complete a task, while health marketing is a top-down approach based on professionals’ idea [16]. Three weeks following the intervention, 624 participants reported HIV test uptake and test results through text messaging, while 97 were lost to follow-up (Additional file 1: Figure S1). Honorariums (~8 USD phone card) were given to those who responded to the messaging.

Bivariable and multivariable logistic regression were used to identify factors associated with reported HIV testing at follow-up, which took place three-week after intervention. The factors examined were: socio-demographics (age, ethnicity, marital status, high education level attained, being student, annual income level and residing in city or countryside), sexual behavior (group sex defined as >2 persons engaged in sexual activity, and sex for money) in the past 12 months, history of suffering intimate partner violence (IPV) by their current male sexual partner and type of IPV experienced (details of IPV published elsewhere [17]), history of sexually transmitted infections (STI) testing, pre- and post-intervention intention of HIV testing in the next year and type of intervention received (health marketing or crowdsourced video). In multivariable logistic regression, besides the type of intervention video received, the following variables were explored as potential confounders: student status, being adolescent (≤19 year-old [18]), married and annual income >9677USD. If there was >10% change between crude odds ratio (OR) and adjusted odds ratio (aOR) in the multivariable model with a confounder, we kept the confounder in the model. We have also examined the factors associated with reported HIV testing results (positive vs negative) in bivariate analyses.

To account for the clustering of HIV testing which might exist in 32 provincial-level administrative divisions (named as provinces hereafter), we performed binomial multilevel models using R 3.2.1 lme4 package. In the model, MSM (level 1) were nested by provinces (level 2). Empty multilevel models were developed to examine the homogeneity of outcomes across provinces. If heterogeneity existed, explanatory multilevel models were performed separately to include variables at individual level (same as those factors listed in logistic regression models) and province level (retrieved from China Statistical Yearbook 2014 http://www.stats.gov.cn/tjsj/ndsj/2014/indexch.htm). Provincial level factors included population size, number of males aged 15 or above, size and proportion of urban population, total and per capita gross regional product of the province. Provincial heterogeneity was tested by median odds ratio (MOR), with 1 denoting the absence of heterogeneity and >1 for higher heterogeneity [19].

## Results

### Baseline characteristics

All never-tested MSM responding to follow-up message (*n* = 624) were included in this study. At baseline, the median age was 22 years old (interquartile range (IQR) = 20–26) and half of the men were students (Table 1). A majority were not married (90%), had received at least high school education (72% diploma or above), earned below 9677 USD in a year (86%) and were living in a city instead of rural area (87%). In the past 12 months, 7% of men had group sex and 5% had sex for money. A total of 286 (46%) intended to test for HIV in the following year before intervention, and the figure increased to 388 (62%) after intervention.

**Table 1:** Characteristics of never-tested MSM in 2014 in China (*n* = 624)

	Frequency	%
Socio-demographics		
Age		
> 19 years old	494	79%
≤ 19 (adolescent) years old	130	21%
Ethnicity^a^		
Non-Han (minority and non-Chinese)	46	7%
Han	575	93%
Currently married	65	10%
Highest education level		
Diploma or above	449	72%
High school or below	175	28%
Currently a student	300	48%
Annual income		
≤ 9677USD	536	86%
> 9677USD	88	14%
Residing area^#^		
City	543	87%
Countryside	81	13%
Sex behaviour (past 12 months)		
Group sex	43	7%
Sex for money	33	5%
History of sexually transmitted infections testing	68	11%
Pre-intervention HIV testing intention		
not to test in the next year	338	54%
would test within the next year	286	46%
Post-intervention HIV testing intention		
not to test in the next year	236	38%
would test within the next year	388	62%
Tested for HIV at follow-up		
No	399	64%
Yes:	225	36%
*Reported to be HIV positive among MSM self-reported HIV testing at follow-up*	69	31%

^a^3 missing values

^#^residing area refers to the self-reported description on their current residential area, either city (urban area) or countryside (rural area)

### Factors associated with HIV testing at follow-up

Among 624 MSM, 225 (36%) self-reported HIV testing, of which 69 claimed to have been tested HIV positive. The HIV prevalence ranged between 11% (69/624, assuming those non-tested were HIV negative) and 31% (69/225). Comparing with HIV negative respondents, reported HIV positive respondents were more likely to be adolescent (HIV negative: 17% vs HIV positive: 29%; OR = 1.95, 95% C.I. = 1.003–3.79) and with lower education level (HIV negative: 23% vs HIV positive: 48%, OR = 3.06, 95% C.I. = 1.67–5.58). Other characteristics, including other socio-demographics, sexual behavior, experience of IPV and history of STI testing, were not significantly different between reported HIV positive and negative respondents (results not shown).

In multivariable logistic regression with confounders of intervention type and age (continuous variable), students were less likely to have undergone HIV testing than non-students (aOR = 0.69, 95% C.I. = 0.47–0.99) (Table 2). Adjusted by intervention type, men ever tested for STIs (aOR = 2.17, 95% C.I. = 1.31–3.61) were more likely to test for HIV. In addition, HIV testing intention before intervention (aOR = 2.39, 95% C.I. = 1.71–3.34) and after intervention (aOR = 2.94, 95% C.I. = 2.03–4.26) were positively associated with HIV testing at follow-up, and with higher odds for post-intervention testing intention.

**Table 2:** Association of MSM’s characteristics with HIV testing at follow-up (*n* = 624, unless otherwise specified)

	Tested for HIV at follow-up		N	Multivariable logistic regression^ϕ^	
	n	%		aOR	(95% C.I.)
Socio-demographics					
Age group					
> 19 years old	178	36%	494		*ref*
≤ 19 (adolescent) years old	47	36%	130	1.00	(0.67–1.5)
Ethnicity^a^					
Non-Han (minority and non-Chinese)	10	22%	46		*ref*
Han	213	37%	575	2.12	(1.03–4.36)*
Currently married					
No	196	35%	559		*ref*
Yes	29	45%	65	1.51	(0.9–2.53)
Highest education level					
Diploma or above	156	35%	449		*ref*
High school or below	69	39%	175	1.22	(0.85–1.75)
Currently a student					
No	127	39%	324		*ref*
Yes	98	33%	300	0.69	(0.47–0.99)*β
Annual income					
≤ 9677 USD	190	35%	536		*ref*
> 9677 USD	35	40%	88	1.20	(0.76–1.9)
Residing area^#^					
City	191	35%	543		*ref*
Countryside	34	42%	81	1.33	(0.83–2.14)
Sex behavior (past 12 months)					
Group sex					
No	204	35%	581		*ref*
Yes	21	49%	43	1.79	(0.96–3.34)
Money for sex					
No	205	35%	591		*ref*
Yes	20	61%	33	2.97	(1.44–6.1)*
Experience of intimate partner violence^b^					
Any type of violence					
No	64	35%	183		*ref*
Yes	42	57%	74	2.44	(1.4–4.23)*
Hit you or thrown objects at you					
No	84	38%	219		*ref*
Yes	22	58%	38	2.19	(1.09–4.42)*
Destroyed your property					
No	95	40%	238		*ref*
Yes	11	58%	19	2.05	(0.8–5.3)
Threatened to stop helping you with money or housing					
No	96	39%	245		*ref*
Yes	10	83%	12	7.68	(1.64–35.9)*
Threatened to harm you and someone you care for					
No	91	38%	238		*ref*
Yes	15	79%	19	6.01	(1.93–18.72)*
Threatened to reveal your sexuality					
No	79	37%	213		*ref*
Yes	27	61%	44	2.68	(1.37–5.23)*
History of STI testing					
No	189	34%	556		*ref*
Yes	36	53%	68	2.17	(1.31–3.61)*
HIV testing intention					
At baseline					
Not to test in the next year	91	27%	338		*ref*
Would test within the next year	134	47%	286	2.39	(1.71–3.34)*
After intervention					
Not to test in the next year	51	22%	236		*ref*
Would test within the next year	174	45%	388	2.94	(2.03–4.26)*
Type of HIV testing video watched					
Health marketing	114	37%	307		*ref*
Crowdsourcing	111	35%	317	crude OR = 1.10	(0.79–1.52)

n-number of men tested for HIV at followup; %-proportion of men tested for HIV at followup

^ϕ^adjusted by the type of intervention received (health marketing video as 0, crowdsourced video as 1) in multivariable logistic regression model

β adjusted by both type of intervention received and adolescent in multivariable logistic regression model

^a^3 missing; ^b^367 missing

^#^residing area refers to the self-reported description on their current residential area, either city (urban area) or countryside (rural area)

*p-value < 0.05

Risky sexual behaviors of sex for money (aOR = 2.97, 95% C.I. = 1.44–6.10) were more likely to be associated with HIV testing. Among 257 men responding to questions related to IPV (the rest not responded to the question), 29% self-reported any type of IPV experience, and the latter were also more likely to test for HIV (aOR = 2.44, 95% C.I. = 1.40–4.23). Specific types of IPV, including being hit or thrown objects, threatened to stop financial help, threatened to harm the person or persons they care for, and threatened to reveal their sexuality were positively associated with HIV testing at follow-up.

**Fig. 1:**
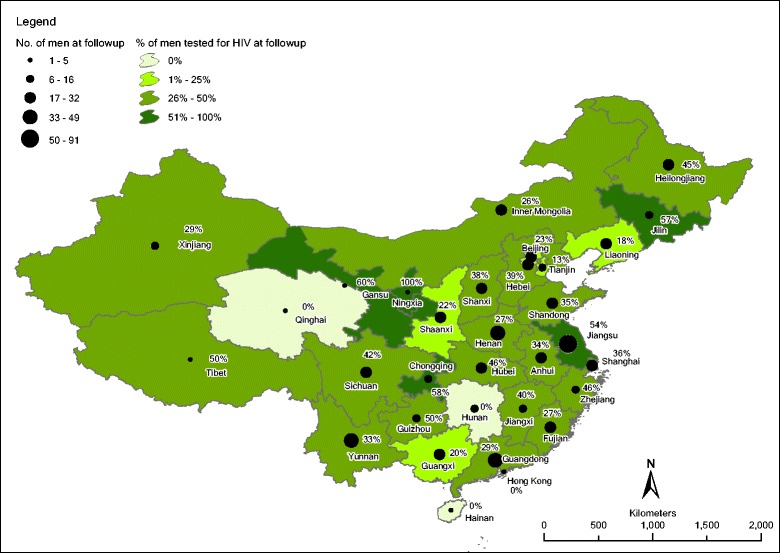
Spatial distribution of number of men at follow-up (*n* = 624) denoted by circle size and proportion of HIV testing at follow-up denoted by province area in graduated color (classified by natural break)

Geographically, the median proportion of testing across provinces was 35% (IQR = 25%–46%, *n* = 26), excluding 6 provinces with ≤5 men in this study. (see Fig. 1 for geographic distribution) Moderate heterogeneity of HIV testing rate at follow-up in 32 provinces was observed with MOR = 1.34. In explanatory multilevel models, having sex for money (aOR = 2.88, 95% C.I. = 1.38–6.01), history of STI testing (aOR = 2.11, 95% C.I. = 1.26–3.55) and intention of HIV testing at baseline (aOR = 2.38, 95% C.I. = 1.69–3.33) and after intervention (aOR = 2.90, 95% C.I. = 2.00–4.22) remained significant positive predictors of HIV testing. However, association of neighborhood effect of province-level factors such as population size or gross regional product with HIV testing was not observed.

## Discussion

In this study, we found that 36% of MSM without previous HIV testing underwent HIV testing after an online intervention. The prevalence of self-reported HIV infection ranged from 11% to 31%. The lower bound estimation was higher than the national HIV prevalence study among MSM [3]. The lower estimated prevalence in the previous study might be contributed by lower risk between HIV tests among MSM with regular testing behavior. This study focused on the factors significantly associated with first-time HIV testing following an online intervention. Demographically, students had a lower HIV testing rate while men with more risky behaviors had a higher HIV testing rate. Previous studies have examined HIV testing mass media interventions [20]. Several studies identified subgroups not effectively reached by such interventions [8, 20]. Our study expands the literature by identifying the characteristics of MSM who were more likely to undergo HIV testing following online intervention in a middle-income country, and examining spatial variation in effects.

Geographically, moderate provincial variation of testing rate at follow-up was found. Moderate provincial variation towards HIV testing action was probably due to the variation of local HIV testing facilities, which we however do not have the data to fit in the model here. Spatial barriers of service utilization and spatial variation in HIV testing rate can be reduced by decentralization of testing sites, as observed in Mozambique [21]. With the expansion of HIV testing services in China in recent years, [2] smaller variation of provincial HIV testing rate is expected.

Though the proportion of men with risky sexual behaviors (having sex for money) was small (7% of never-tested men), they were more likely to have HIV testing at follow-up. A high proportion of high risk MSM (38% of those having group sex and 45% of those having sex for money) claimed to be HIV positive, though these risk behaviors were not significantly associated with HIV positive status. Conversely, men without high risk sexual behaviors were less likely to go for HIV testing, probably because of their low perceived risk of infection, which is consistent with other studies for never-tested MSM [22, 23]. On the other hand, IPV experience was found to be significantly associated with high risk behaviors of group sex [17] and having sex for money [17, 24]. In our study, 29% (74 out of 257) of never-tested men at follow-up had experienced IPV. We found that several types of IPV were positively associated with post-intervention testing, which was consistent with a previous study in the U.S. showing similar associations [25]. They were also positively associated with HIV diagnosis [17].

Half of the study population was students. They were less likely to have HIV testing. Even though no significant difference of testing rate was found by age group and age (continuous variable), the students were apparently younger (median age = 20, IQR = 19–22 years old) than non-students (median age = 25, IQR = 22–31). Other studies found that younger MSM were less likely to have been tested for HIV, probably because of their fear of testing in healthcare or local office settings [26, 27]. Of note, this subgroup (young MSM and/or students) could be a targeted population for HIV prevention and control. This is because sexual behaviors such as group sex and having sex for money among students were not lower than non-students in our study (statistical results not shown). In addition, among those underwent HIV testing, adolescent and those with lower education level were more likely to be HIV positive (self-reported). It is possible that online intervention could reach a group of undiagnosed young adults living with HIV in China. With low post-intervention testing rate among students, other types of testing interventions such as school-based interventions may be needed to complement online intervention.

Our analysis has several limitations. Frist, this secondary analysis study did not allow us to prove the causal relationship between pre- and post-intervention HIV testing, and between testing intention and post-intervention testing action. However, the significant association of pre- and post-intervention testing intention with post-intervention testing action could show some linkage. Second, the sample size of this study was relatively small for performing a multilevel model nested by provinces, and therefore variables of ethnicity and IPV were excluded in the multilevel models. Third, like most other MSM studies, we used convenience sampling for recruiting MSM online as the overall sampling frame was unknown. Even though the internet is not limited by spatial distance, the coverage of recruited men was higher in the province hosting the website portal, which might introduce selection bias. We used multilevel models to account for possible heterogeneity. Fourth, HIV testing and test results were self-reported, which could not be validated. However, we believe that the social desirability bias was low because there was no additional incentive for undergoing HIV testing. We also used participants’ mobile phone numbers to prevent duplicate responses.

## Conclusions

The proportion of intent to test of never-tested men rose from 46% at baseline to 62% after mass media intervention, with HIV testing reported by 36% at follow-up. Men who intended to test before and after intervention were more likely to test at follow-up. Men with high risk sexual behaviors, IPV experience and STI testing experience had higher preference for HIV testing. However, as student MSM were less likely to undergo testing even after intervention, further research is needed to enhance HIV testing and explain why adolescents were more likely to be HIV positive once tested. Possible future interventions include online tools complemented by conventional school-based interventions.

## Additional file

Additional file 1: Figure S1. Study layout. (PDF 170 kb)

## Abbreviations

aOR: adjusted odds ratio
IPV: Intimate partner violence
IQR: Interquartile range
MOR: Median odds ratio
MSM: Men who have sex with men
OR: Odds ratio
STI: Sexually transmitted infections
