# Supplementary figures and images for: MSM HIV testing following an online testing intervention in China
Source: BMC Infect Dis. 2017 Jun 19;17:437. doi: 10.1186/s12879-017-2546-y (PMC5477382; doi:10.1186/s12879-017-2546-y)

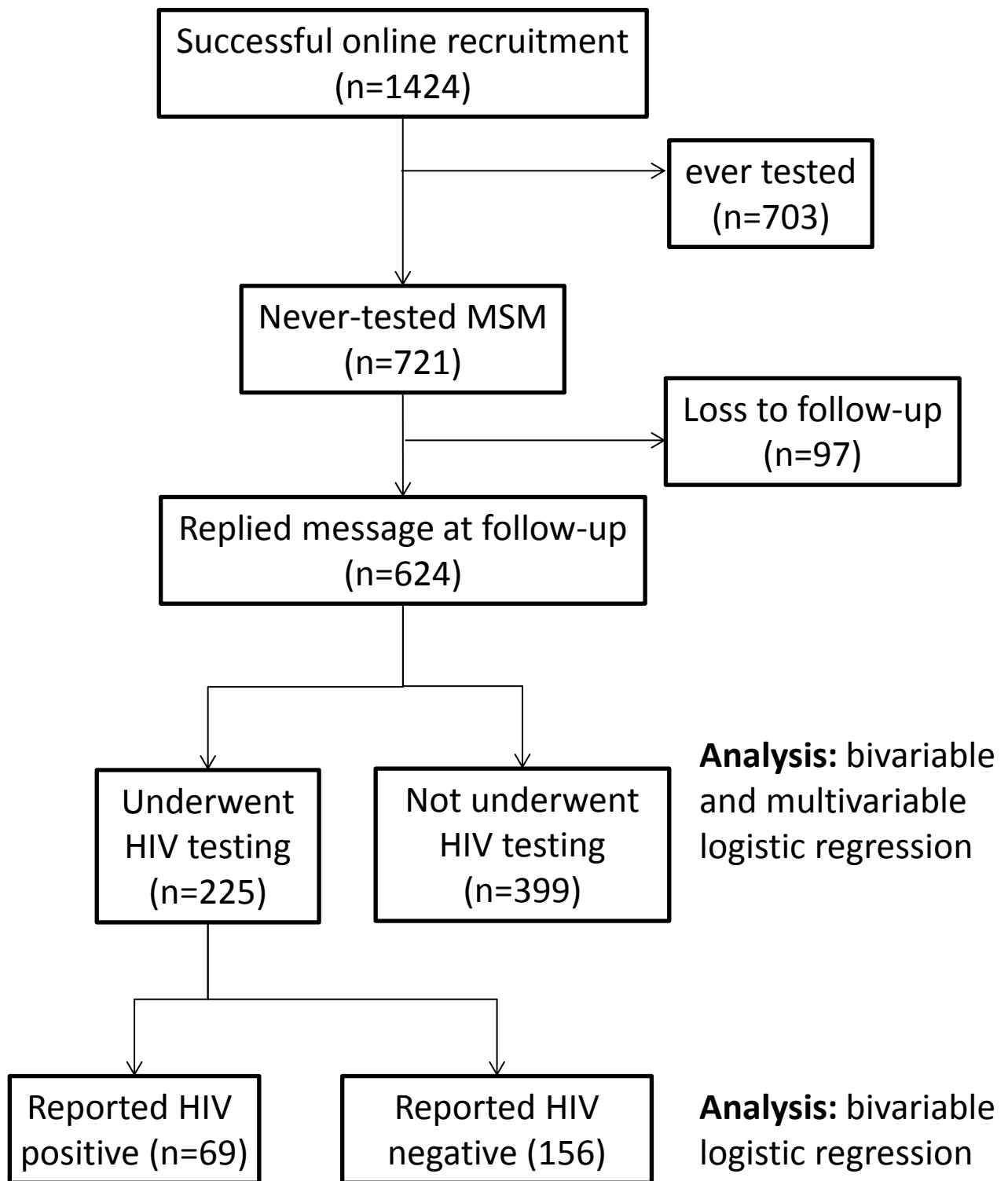

Supplement: Additional file 1: Figure S1. — Study layout. (PDF 170 kb) [file 12879_2017_2546_MOESM1_ESM.pdf]
